# 高效液相色谱紫外等吸收波长法快速测定秦皮中秦皮甲素和秦皮乙素

**DOI:** 10.3724/SP.J.1123.2023.03018

**Published:** 2023-08-08

**Authors:** Zhengming QIAN, Mengqi WU, Guoying TAN, Liling JIN, Ning LI, Juying XIE

**Affiliations:** 1.湘南学院医学影像检验与康复学院,湖南 郴州 423000; 1. College of Medical Imaging Laboratory and Rehabilitation, Xiangnan University, Chenzhou 423000, China; 2.东莞市东阳光冬虫夏草研发有限公司,广东 东莞 523850; 2. Dongguan HEC Cordyceps R&D Co., Ltd., Dongguan 523850, China; 3.云南中医药大学中药学院,云南 昆明 650500; 3. College of Traditional Chinese Medicine, Yunnan University of Chinese Medicine, Kunming 650500, China

**Keywords:** 高效液相色谱, 等吸收波长, 核壳型色谱柱, 秦皮甲素, 秦皮乙素, high performance liquid chromatography (HPLC), equal absorption wavelength (EAW), core-shell chromatographic column, aesculin, aesculetin

## Abstract

研究建立了一种快速、环保和节约对照品的秦皮化学成分含量测定方法。秦皮样品采用25%(v/v)乙醇水溶液超声提取5 min。样品提取溶液采用Poroshell 120 EC-C_18_色谱柱(50 mm×4.6 mm, 2.7 μm)进行分析,0.1%甲酸水溶液-乙腈(94∶6, v/v)等度洗脱,流速1.5 mL/min。采用紫外分光光度仪对等浓度的秦皮甲素和秦皮乙素进行紫外光谱扫描,初步筛选得到2个对照品的等吸收波长,并用高效液相色谱仪对筛选得到的等吸收波长进行确证,从而获得2个对照品的等吸收检测波长341 nm。新建立的高效液相色谱紫外等吸收波长法的方法验证结果显示,秦皮甲素和秦皮乙素在各自的浓度范围内具有良好的线性关系(*r*=1.0000),检出限和定量限分别为1.5 μmol/L和3.0 μmol/L,平均加标回收率分别为99.0%(RSD=1.4%)和97.5%(RSD=0.9%)。通过比较高效液相色谱紫外等吸收波长法和传统高效液相色谱外标法对10批秦皮样品中秦皮甲素和秦皮乙素的含量测定结果,显示两种方法测定结果一致。此外还采用*t*检验对该方法与《中国药典》2020版方法的含量测定结果进行比较,对比结果表明两种测定方法无显著性差异(*P*>0.05)。该方法耗时10 min,有害溶剂消耗0.5 mL,只使用1个对照品,具有快速、简便、环保和对照品消耗少的特点,适用于秦皮中秦皮甲素和秦皮乙素的含量测定,为秦皮质量评价的技术提升提供了依据。

## Abstract (en)

Fraxini Cortex is a traditional Chinese herbal medicine that has been used for thousands of years to treat dampness-heat diarrhea, dysentery, red or white vaginal discharge, painful swelling or redness of the eyes, and nebula. It contains various chemical components, including coumarins, iridoids, phenolic acids, and flavonoids. Coumarins are important active ingredients in Fraxini Cortex and have antibacterial, anti-inflammatory, antioxidant, antitumor, and antiviral activities. Aesculin and aesculetin are two major coumarin components of Fraxini Cortex that are widely used in its quality evaluation. Previous HPLC methods for determination of aesculin and aesculetin present several limitations, such as long analysis times and high solvent and reference compound consumption.

In this study, a rapid, eco-friendly and cost saving HPLC method for the determination of aesculin and aesculetin in Fraxini Cortex was established by using the core-shell column and equal absorption wavelength (EAW). Different factors influencing the extraction process, such as the extraction solvent, temperature, and time, were assessed to obtain the optimal extraction conditions. The results showed that Fraxini Cortex samples could be well extracted by ultrasonic extraction for 5 min with a 25% ethanol aqueous solution. A core-shell column was used, and different mobile phases and flow rates were investigated to obtain the best rapid-HPLC separation conditions. The optimized HPLC conditions were as follows: a Poroshell 120 EC-C_18_ column (50 mm×4.6 mm, 2.7 μm), acetonitrile-0.1% formic acid aqueous solution (6∶94, v/v) as the eluent, a flow rate of 1.5 mL/min, and a column temperature of 25 ℃.

The EAW of aesculin and aesculetin was a key factor in their determination using a single reference compound. EAW selection was performed in two steps. First, the UV spectra of two equimolar concentrations of the reference compounds (aesculin and aesculetin) were compared to determine the EAW of the two analytes. The EAW results were then verified by the HPLC analysis of the reference compound solutions. The final EAW of aesculin and aesculetin was 341 nm. The determination of aesculin and aesculetin using only one reference compound (i. e., aesculin) was achieved by HPLC-UV at this EAW.

The newly developed HPLC method revealed a good linear relationship between the two target analytes (*r*=1.0000). The limits of detection (LODs) and limits of quantification (LOQs) were 1.5 μmol/L and 3.0 μmol/L, respectively, and the average recoveries of aesculin and aesculetin were 99.0% and 97.5%. The stabilities of the sample solutions were examined, and the two analytes demonstrated good stability for 24 h. The contents of the target analytes in 10 batches of Fraxini Cortex were determined using the proposed EAW method and the classic external standard method (ESM), and comparable concentrations were obtained. The contents of aesculin and aesculetin in the 10 batches of Fraxini Cortex were 0.26%-2.80% and 0.11%-1.47%, respectively. A *t*-test was conducted to compare the results of the proposed EAW technique with those obtained via the method reported in the Chinese Pharmacopoeia, and no significant difference between the two assay methods was noted (*P*>0.05). Comparison of the newly established EAW method with those reported in the literature revealed that our method required only 10 min to complete and used as little as 0.5 mL of the solvent and only one standard. Therefore, the developed EAW method is a rapid, simple, eco-friendly, and cost-effective analytical method that is suitable for the determination of aesculin and aesculetin in Fraxini Cortex and its related products. The proposed technique is an improved method for determining aesculin and aesculetin and contributes to the enhancement of the quality evaluation of Fraxini Cortex.

秦皮为传统中药材,在《神农本草经》中就有记载,其具有清热燥湿、收涩止痢、止带和明目的功效^[1]^。现代药学研究表明秦皮含有多类化学成分,包括香豆素、环烯醚萜、酚酸和黄酮等^[2,3]^。香豆素类成分是秦皮的主要活性成分,具有抗炎、抗氧化、抗肿瘤、抗菌和抗病毒的药理活性^[4⇓-6]^。其中秦皮甲素和秦皮乙素为秦皮药材中的主要香豆素成分,其具有抗氧化、抗肿瘤和抗糖尿病等作用^[7⇓-9]^,被广泛应用于秦皮药材的质量评价。前期报道采用高效液相色谱^[10⇓-12]^、毛细管电泳^[13]^、胶束电动色谱^[14]^和荧光光谱^[15]^等技术建立了秦皮中秦皮甲素和秦皮乙素含量的测定方法。其中高效液相色谱法是《中国药典》规定的秦皮药材含量测定的方法,同时也是秦皮产业中应用最多的方法。文献报道的秦皮高效液相色谱含量测定方法存在分析时间长、对照品消耗多的问题。叶皓等^[10]^采用超声法制备样品,提取时间8.5 min,液相色谱分析时间63 min;赵重博等^[11]^采用回流法制备样品,提取时间60 min,液相色谱分析时间60 min。以上方法的总分析时间均在60 min以上,同时也都需要2个对照品进行定量。

高效液相色谱-紫外检测分析定量原理为通过色谱柱对化合物进行分离,紫外检测器依据分析物特定波长的响应进行含量测定。前期方法为了确保分析方法的灵敏度,通常采用秦皮甲素和秦皮乙素的最大吸收波长作为检测波长。秦皮甲素和秦皮乙素在最大吸收波长下紫外响应不一样,因此需要采用校正因子才能实现单一对照品对多个化合物的定量^[16]^。如果采用秦皮甲素和秦皮乙素具有相同紫外吸收的波长作为检测波长,就有可能实现对秦皮甲素和秦皮乙素进行含量测定时采用单一对照品,并且不需要使用校正因子进行换算。核壳色谱柱具有柱效高、柱压低和分析速度快的优点^[17,18]^,是一种适用于中药产业化快速分析检测的液相色谱柱。区别于普通硅胶色谱柱的全多孔填料,核壳型色谱填料是由无孔硅胶核及外层包裹的多孔核壳层组成的一种表面多孔微球,其多孔的核壳层使溶剂扩散路径更短、理论塔板高度更小,因而具有更高的柱效。同时核壳填料具有更好的通透性,背压低,可使用更高的分析流速。它可以在常规液相色谱上实现类似超高效液相色谱的快速分析,在不增加产业设备投入的情况下,可以提高产业化检测效率和降低检测成本,目前已被应用于冬虫夏草、蛹虫草、人参和延胡索等多种中药材的液相色谱分析^[19⇓⇓-22]^。本研究采用核壳色谱柱和紫外等吸收波长的技术建立了单一对照品直接定量秦皮中秦皮甲素和秦皮乙素的快速HPLC分析方法,该研究为秦皮质量评价的技术提升提供了依据。

## 1 实验部分

### 1.1 仪器、试剂及材料

Agilent 1260 Infinity二代高效液相色谱仪、Agilent 1260 Infinity一代高效液相色谱仪、Cary 60型紫外-可见分光光度计(美国Agilent公司), XPE205DR型十万分之一天平(美国Mettler Toledo公司), Milli-Q型超纯水仪(德国Millipore公司), P300H型超声仪(德国ELMA公司)。

无水乙醇(分析纯)购自成都科隆化学品有限公司,甲酸(色谱纯)购自上海阿拉丁试剂有限公司,乙腈(色谱纯)购自上海阿达玛斯试剂有限公司,秦皮甲素(纯度99.77%)、秦皮乙素(纯度99.94%)均购自成都普思生物科技有限公司。

秦皮饮片样品10批(S1~S10),均经钱正明主任中药师鉴定为木犀科植物白蜡树*Fraxinus chinensis* Roxb.的干燥枝皮或干皮,其中S1~S3来自康美药业股份有限公司,S4来自岭南中药材饮片有限公司,S5~S10为市售。

### 1.2 对照品溶液配制

精密称取秦皮甲素和秦皮乙素各20.18 mg,分别加无水乙醇溶解定容至10 mL,混匀,得到浓度分别为5917 μmol/L和11320 μmol/L的对照品储备液。再精密移取秦皮甲素、秦皮乙素对照品储备液,加25%(v/v)乙醇水溶液稀释得到浓度分别为147.9 μmol/L和148.2 μmol/L的混合对照品工作液。

### 1.3 供试品溶液制备

秦皮饮片粉碎,过三号筛,精密称取约50 mg,置于50 mL离心管中,加入25%(v/v)乙醇水溶液10 mL, 50 ℃超声(功率380 W,频率37 kHz)提取5 min,摇匀,吸取上清液,过0.22 μm有机滤膜,待测。

### 1.4 色谱条件

色谱柱为Agilent Poroshell 120 EC-C_18_(50 mm×4.6 mm, 2.7 μm),柱温25 ℃,流动相为乙腈-0.1%(v/v)甲酸水溶液(6∶94, v/v),等度洗脱,流速1.5 mL/min,进样量1 μL,检测波长341 nm,带宽4 nm。

## 2 结果与讨论

### 2.1 实验条件优化

#### 2.1.1 提取条件优化

超声提取是一种简便高效的中药材样品提取方法,前期文献采用该方法以甲醇-水为溶剂对秦皮中的化合物进行提取^[10]^。本实验为了获得一种绿色高效的样品制备方法,选择绿色溶剂乙醇和水作为提取溶剂,并进一步对不同体积分数的乙醇水溶液、提取溶剂用量、提取时间和提取温度进行了单因素考察,每个考察条件均平行测定2份。首先比较了不同体积分数(0、25%、50%、75%和100%)的乙醇水溶液的提取率,结果发现25%及以上体积分数的乙醇水溶液作提取溶剂时秦皮甲素和秦皮乙素提取率较好(图1a),考虑到25%乙醇水溶液有机溶剂用量较少,以及其极性与流动相较为接近不易产生溶剂效应,故选择25%乙醇水溶液作为提取溶剂。

**图1:**
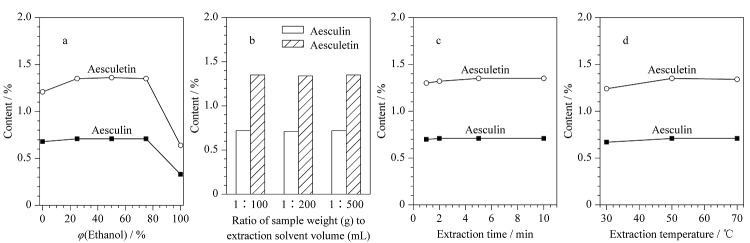
不同提取条件对秦皮甲素、秦皮乙素含量的影响

通过比较不同提取药材质量(g)与提取溶剂体积(mL)的比例(1∶100、1∶200、1∶500)对秦皮甲素和秦皮乙素提取率的影响,发现提取药材质量与提取溶剂体积比对目标化合物的提取率影响不大(图1b),最后采用1∶200进行提取。通过样品超声时间(1、2、5、10 min)的考察发现超声提取5 min即可完成分析物的充分提取(图1c)。通过不同提取温度(30、50、70 ℃)的比较发现50 ℃和70 ℃均具有较好的提取率(图1d),考虑到50 ℃能耗较低,因此选择50 ℃作为提取温度。综上所述,本实验提取方法为取秦皮粉末约50 mg,置于50 mL离心管中,加入10 mL 25%乙醇水溶液,50 ℃超声5 min。

#### 2.1.2 色谱条件的优化

前期有关秦皮定量分析的报道^[10⇓-12]^显示,所采用的流动相体系中有机相主要为甲醇、乙腈,水相主要为甲酸水溶液、磷酸水溶液;色谱柱多采用普通C_18_全多孔硅胶色谱柱,分析时间为25~63 min。核壳型色谱柱具有通透性好、背压低等优势,与全多孔填料相比,该填料可显著缩短分析时间,改善分离效果,提高灵敏度。本研究为了实现快速色谱分离,采用了Poroshell 120 EC-C_18_核壳色谱柱(50 mm×4.6 mm, 2.7 μm)。同时对水-甲醇、0.1%磷酸水溶液-甲醇、水-乙腈、0.1%甲酸水溶液-乙腈4种流动相体系进行了比较,结果显示,流动相为0.1%甲酸水溶液-乙腈时,目标色谱峰分离效果及峰形最优。通过比较不同的流速(1.0 mL/min和1.5 mL/min),发现在1.5 mL/min的流速下分析时间明显缩短且分离度(*R*)符合药典要求(*R*>1.5),故选择流速为1.5 mL/min。本方法可实现对秦皮的快速分析,但与前期秦皮含量测定研究^[10⇓-12]^中使用的普通C_18_全多孔硅胶色谱柱相比,核壳型色谱柱载样量较小^[23,24]^,导致一般进样体积较小,同时色谱技术相对先进,核壳柱的价格也比普通全多孔色谱柱要贵一些,随着未来核壳型色谱柱技术的发展以及应用市场的普及,核壳柱载样量低和价格贵的问题相信也一定会得到改善。

#### 2.1.3 等吸收检测波长的筛选

文献报道方法^[10⇓-12]^通常采用外标法对秦皮中的秦皮甲素和秦皮乙素进行含量测定,需要用到两种对照品进行定量,其存在对照品消耗多和对照品溶液配制繁琐的问题。等吸收波长检测法是通过找到两个对照品溶液具有相同紫外吸收的波长作为高效液相色谱检测波长。在该波长下,两种对照品溶液的紫外响应相同,以其中任何一个物质作为对照品即可实现对两种分析物的含量测定,同时也不需要额外的校正因子进行换算,真正实现了单一对照品对两种分析物的同时测定。

为了获得秦皮甲素和秦皮乙素的紫外等吸收波长,采用紫外-可见分光光度计对秦皮甲素(20.13 μg/mL)和秦皮乙素(20.17 μg/mL)的对照品溶液进行扫描。由图2a可知,等质量浓度的秦皮甲素和秦皮乙素对照品溶液在紫外光谱中不存在等吸收波长。对比2个分析物的结构发现秦皮甲素和秦皮乙素具有一样的紫外基团,但秦皮乙素比秦皮甲素少一个糖基团,其相对分子质量较小,故在相同质量浓度溶液中,秦皮乙素比秦皮甲素的浓度更高,所以在等质量浓度情况下秦皮乙素的紫外吸收始终高于秦皮甲素。因此实验改用等浓度的秦皮甲素(29.58 μmol/L)和秦皮乙素(29.64 μmol/L)对照品溶液进行紫外波长扫描测试。通过比较2种分析物的紫外光谱图(图2b)发现226 nm和342 nm是其紫外响应较高的2个等吸收波长。采用HPLC对样品溶液进行分析,比较测试溶液在226 nm处和342 nm处的色谱图,发现秦皮甲素和秦皮乙素在342 nm处的色谱图整体出峰情况和基线噪声均优于226 nm处的色谱图,因此选择342 nm作为候选HPLC等吸收测定波长,并进行下一步测试。

**图2:**
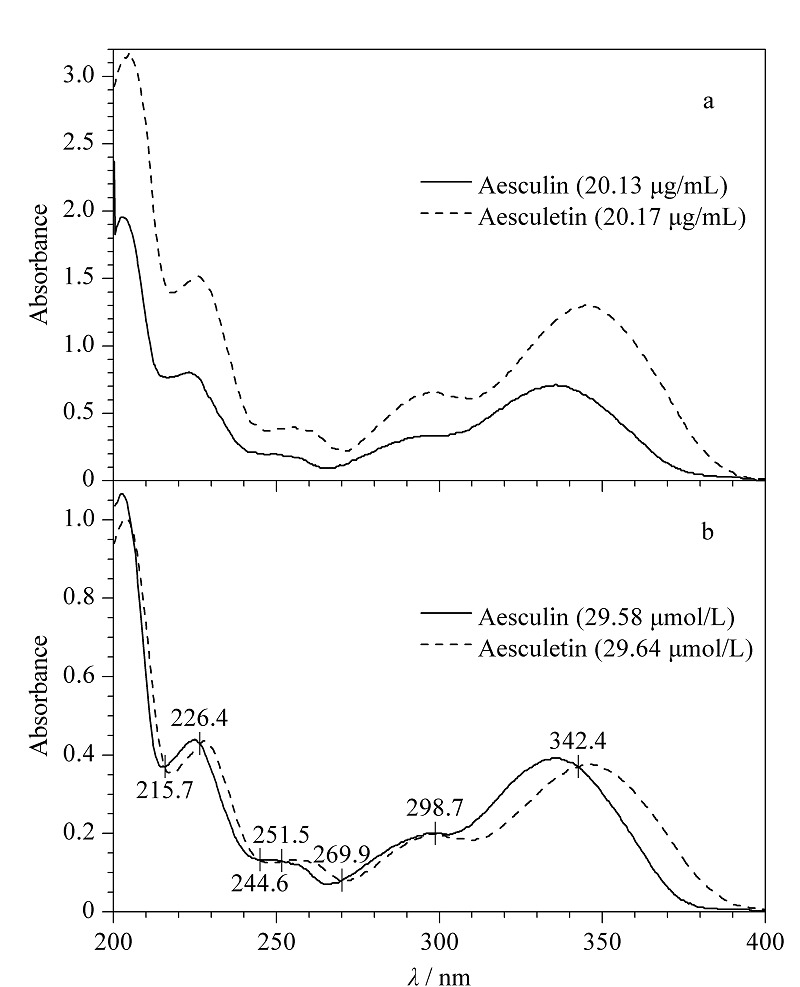
秦皮甲素和秦皮乙素的紫外光谱

为了对候选波长进行确认,实验采用了2个不同分析实验室的不同高效液相色谱仪(实验室1: Agilent 1260 Infinity二代;实验室2: Agilent 1260 Infinity一代)对等浓度的分析物溶液进行分析,以340、341、342、343和344 nm为检测波长,记录不同波长下秦皮甲素和秦皮乙素的峰面积(图3), 2个不同高效液相色谱仪的检测结果均显示,当等吸收检测波长为341 nm时,两个分析物的峰面积最接近,因此选择了341 nm作为HPLC紫外等吸收波长。通过对不同检测带宽(1、2、4、6 nm)进行了比较,发现带宽对秦皮甲素、秦皮乙素峰面积影响不大,最终采用检测带宽为4 nm。

**图3:**
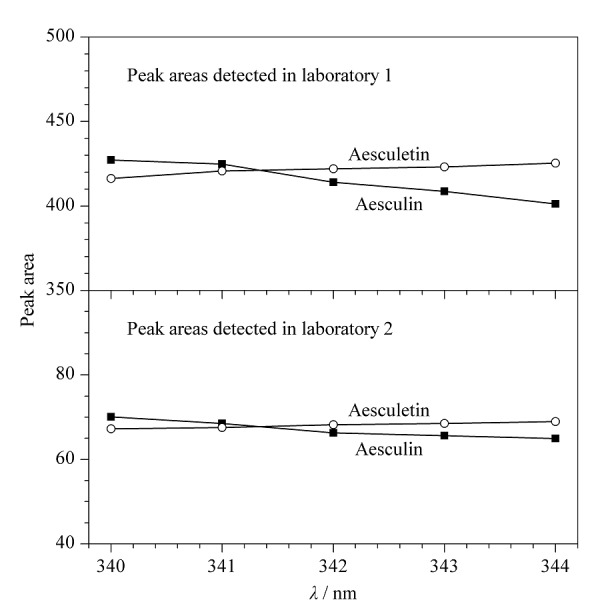
等浓度的秦皮甲素、秦皮乙素对照品溶液在不同检测波长下的峰面积

#### 2.1.4 秦皮乙素色谱峰的定位

按1.3节方法制备供试品溶液,按1.4节色谱条件进样分析,使用2个实验室的不同型号高效液相色谱仪分别平行测定6份,以秦皮甲素为参照峰(S),计算得秦皮乙素相对保留时间均值分别为1.73、1.78, RSD分别为0.29%、0.40%,表明可以将秦皮甲素作为参照峰,用相对保留时间定位秦皮乙素色谱峰。

### 2.2 方法学考察

#### 2.2.1 线性关系、检出限和定量限

按1.2节方法配制秦皮甲素、秦皮乙素对照品储备液,分别用25%(v/v)乙醇水溶液逐级稀释成443.8、295.8、147.9、74.0、14.8 μmol/L系列浓度和444.6、296.4、148.2、74.1、14.8 μmol/L系列浓度的对照品溶液,按1.4节色谱条件进行分析,以各分析物峰面积*Y*为纵坐标,浓度*X*(μmol/L)为横坐标进行线性回归,以信噪比≥3和10的浓度分别确定分析方法的检出限(LOD)和定量限(LOQ)。

结果表明,秦皮甲素和秦皮乙素在各自的线性范围内具有良好的线性关系,相关系数(*r*)=1.0000,方法的LOD为1.5 μmol/L, LOQ为3.0 μmol/L(见表1)。

**表1:** 秦皮甲素和秦皮乙素的线性范围、线性方程、相关系数、检出限和定量限

Compound	Linear range/(μmol/L)	Linear equation	*r*	LOD/(μmol/L)	LOQ/(μmol/L)
Aesculin	14.8-443.8	*Y*=2.7820*X*+1.1814	1.0000	1.5	3.0
Aesculetin	14.8-444.6	*Y*=2.7499*X*+1.7877	1.0000	1.5	3.0

*Y*: peak area; *X*: concentration, μmol/L.

#### 2.2.2 精密度和稳定性

按1.3节方法制备供试品溶液,以同一天6份样品平行测定结果的相对标准偏差评价方法的精密度。结果显示秦皮甲素和秦皮乙素精密度分别为0.80%、0.69%,表明该方法重复性良好。

将供试品溶液置于室温下,于制备后0、2、4、8、12、18、24 h进样测定,测得秦皮甲素和秦皮乙素峰面积RSD值分别为0.29%、0.30%,表明供试品溶液在24 h内稳定。

#### 2.2.3 回收率

选择已知含量的秦皮样品进行加标回收试验,精密称取样品25 mg,添加秦皮甲素、秦皮乙素对照品储备液至样品中,按1.3节方法制备供试品加标溶液,平行测定6份,计算秦皮甲素、秦皮乙素的回收率。结果显示,秦皮甲素的回收率为97.1%~101.1%,秦皮乙素的回收率为96.3%~98.7%,平均回收率分别为99.0%、97.5%, RSD分别为1.4%、0.9%,表明该方法准确度良好。

### 2.3 样品分析

按1.4节色谱条件对空白溶液、混合对照品溶液和样品溶液进行分析,色谱图见图4。10批秦皮样品每批平行测定2份,分别采用外标法和等吸收波长法计算含量。外标法使用秦皮甲素、秦皮乙素作为对照品,分别计算样品中秦皮甲素、秦皮乙素的含量。等吸收波长法使用秦皮甲素作为对照品,计算样品中秦皮乙素的含量,结果见表2。通过计算两种方法的相对误差(RE), RE=(*ω*_EAW_-*ω*_ESM_)/*ω*_ESM_×100%(式中*ω*_EAW_为等吸收波长法测得的含量,*ω*_ESM_为外标法测得的含量),发现10批秦皮样品两种方法测得的秦皮乙素含量结果相对误差在1%以内,表明两种方法无明显差异,等吸收波长法可以用于秦皮中2个分析物的含量测定。HPLC-UV等吸收波长法测得10批秦皮中的秦皮甲素、秦皮乙素含量分别为0.26%~2.80%、0.11%~1.47%,总量为0.37%~2.91%,其中9批样品符合《中国药典》要求(秦皮甲素及秦皮乙素的总量不得少于0.8%)。

**图4:**
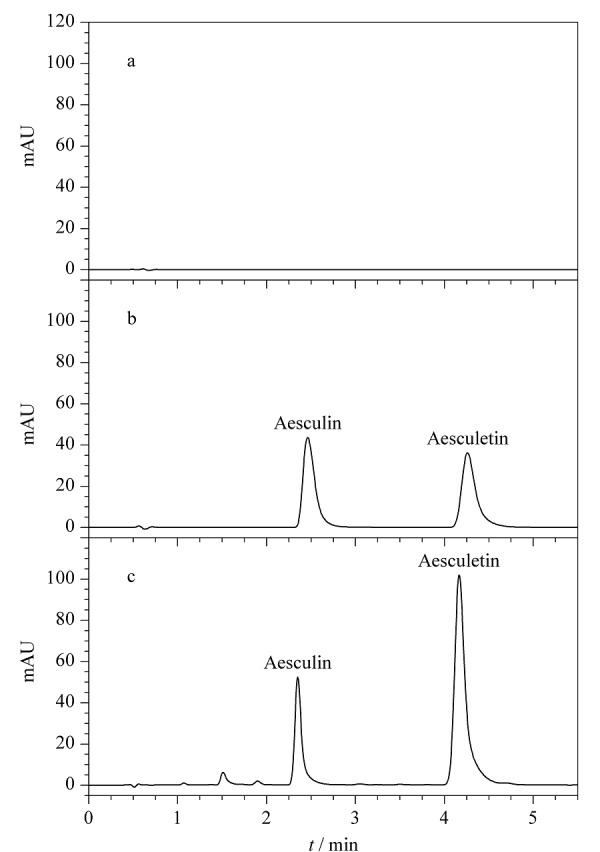
(a)空白溶液、(b)对照品溶液和(c)样品溶液的色谱图

**表2:** 秦皮样品中秦皮甲素和秦皮乙素的含量

No.	Aesculin (ESM)	Aesculetin	
		ESM	EAW
S1	1.22	0.11	0.11
S2	2.11	0.19	0.19
S3	1.86	0.22	0.22
S4	2.14	0.37	0.37
S5	0.77	1.48	1.47
S6	0.83	0.23	0.22
S7	2.80	0.12	0.11
S8	0.68	0.33	0.33
S9	0.41	0.40	0.40
S10	0.26	0.11	0.11

ESM: external standard method; EAW: equal absorption wavelength.

### 2.4 与报道方法比较

秦皮样品分别采用本方法与《中国药典》(2020版)方法进行秦皮甲素和秦皮乙素的含量分析,平行测定6份,样品色谱图见图5。结果显示HPLC-UV等吸收波长法测得秦皮甲素和秦皮乙素的含量为0.77%±0.01%和1.46%±0.01%,药典方法测得秦皮甲素和秦皮乙素含量分别为0.77%±0.01%和1.47%±0.01%。用*t*检验对2组结果进行分析,结果表明两种方法含量测定结果无显著性差异(*P*>0.05)。

**图5:**
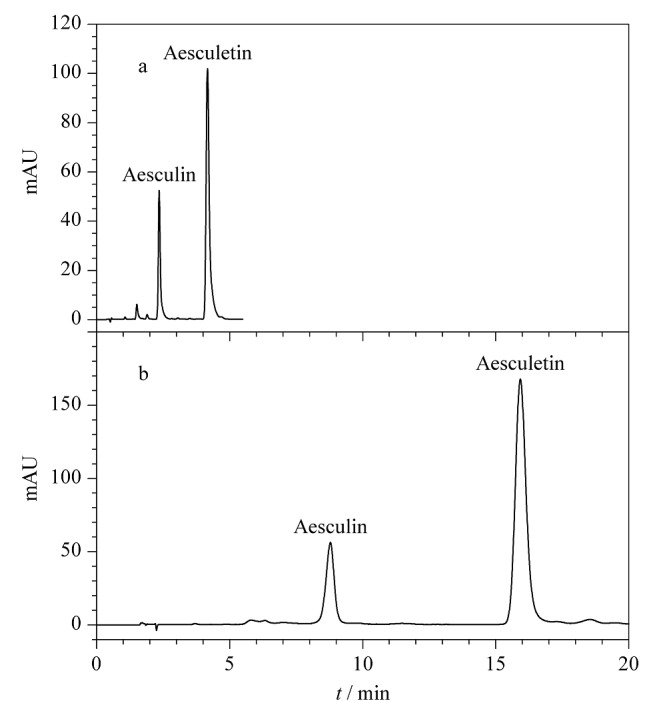
采用(a)本方法和(b)《中国药典》方法时秦皮样品的色谱图

前期文献^[1,10⇓-12]^报道方法多采用外标法和全多孔C_18_色谱柱对秦皮甲素和秦皮乙素进行含量测定(表3),大多方法存在分析时间长(70 min以上)、有机溶剂消耗多(40 mL有害有机溶剂以上)和对照品消耗多(需2个对照品进行定量)的问题。2020年*J Sep Sci*报道了一种绿色的秦皮定量分析方法^[12]^,方法使用低共熔溶剂超声提取,降低了有机溶剂消耗,但仍需较长的分析时间和2个对照品,同时使用的低共熔溶剂非实验室常规提取试剂,制备过程复杂,不利于方法的产业化推广。本方法通过对样品制备条件和色谱分析条件的优化,在常规实验室条件下(普通液相色谱系统和超声装置), 11 min内完成秦皮中秦皮甲素和秦皮乙素的含量测定,同时方法只需要0.5 mL乙腈和1个对照品,大大提升了样品的检测效率,降低了检测成本和环境污染。

**表3:** 本方法与文献报道的比较

No.	Sample extraction				HPLC separation		Harmful solvent	Number of reference compound	Ref.
	Method	Solvent	Time/min		Mobile phase	Time/min			
1	RE	50 mL methanol	60		20 mL acetonitrile-0.1% (v/v) phosphoric acid aqueous solution	20	50.0 mL methanol and 1.6 mL acetonitrile	2	[1]
2	UE	24 mL 94% (v/v) methanol aqueous solution	8.5		50.4 mL methanol-0.1% (v/v) formic acid aqueous solution	63	42.6 mL methanol	2	[10]
3	RE	50 mL methanol	60		60 mL acetonitrile-0.1% (v/v) phosphoric acid aqueous solution	60	50.0 mL methanol and 7.2 mL acetonitrile	2	[11]
4	UE	10 mL synthesized DES containing 20% (v/v) water	30		12.5 mL methanol-0.1% (v/v) formic acid aqueous solution	25	3.8 mL methanol	2	[12]
5	UE	10 mL 25% (v/v) ethanol aqueous solution	5		8.25 mL acetonitrile-0.1% (v/v) formic acid aqueous solution	5.5	0.5 mL acetonitrile	1	this method

HPLC separation time was the end time of chromatogram in literature. RE: reflux extraction; UE: ultrasonic extraction; DES: deep eutectic solvent.

## 3 结论

本研究通过考察秦皮甲素和秦皮乙素的紫外吸收特点,筛选得到了两者的紫外等吸收波长,并在此基础上建立了一种HPLC-UV等吸收波长法测定秦皮中秦皮甲素和秦皮乙素含量的分析方法,真正实现了采用1个对照品对秦皮甲素和秦皮乙素的同时测定。与《中国药典》及文献报道的方法相比,本方法具有快速、简便和环保的优点。该方法的建立为秦皮及其相关产品质量评价的技术提升提供了依据,同时也为其他药材的快速绿色HPLC定量分析方法开发提供了参考。
